# Toileting behavior and urinary tract symptoms among younger women

**DOI:** 10.1007/s00192-017-3319-2

**Published:** 2017-04-05

**Authors:** Johanna Sjögren, Lars Malmberg, Karin Stenzelius

**Affiliations:** 1 Urology Department, Skåne University Hospital, Jan Waldenströms gata 7, SE-205 02 Malmö, Sweden; 2 Faculty of Health and Society, Malmö University, Jan Waldenströms gata 25, SE-205 06 Malmö, Sweden

**Keywords:** Toileting behavior, Lower urinary tract symptoms (LUTS), Voiding, Women, Young women

## Abstract

**Introduction and hypothesis:**

Irregular or infrequent voiding due to avoiding school toilets can contribute to a number of urinary problems among school children. There is, however, a lack of studies on younger women. The aim of this study was to investigate toileting behavior and the correlation to lower urinary tract symptoms (LUTS) among young women (age 18–25 years). A further aim was to validate the Swedish version of the Toileting Behavior scale (TB scale).

**Methods:**

Quantitative descriptive design was used with two questionnaires: the International Consultation on Incontinence Questionnaire Female Lower Urinary Tract Symptoms (ICIQ-FLUTS) and the TB scale, together with six background questions. The questionnaires were distributed in November 2014 to 550 women aged 18–25 years randomly selected from the population register in southern Sweden.

**Results:**

A total of 173 (33%) women responded. Mean age was 21.6 years (range 18–25). The Swedish version of TB scale showed good construct validity and reliability, similar to the original. Most toileting behavior was significantly correlated with LUTS, which were common, as 34.2% reported urgency and 35.9% urine leakage at least sometimes or more often.

**Conclusions:**

LUTS were quite common in this group of young women. Toileting behaviors were also significantly related to urinary tract symptoms. Thus, TB scale was useful in this population, and the translated Swedish version showed good construct validity and reliability.

## Introduction

**Fig. 1:**
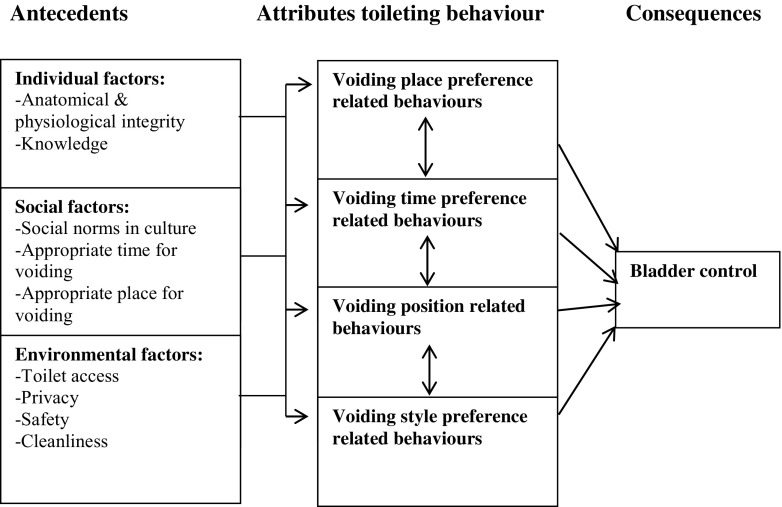
Theoretical framework for women’s toileting behavior (Wang and Palmer [17])

It is well known that irregular or infrequent voiding due to avoiding school toilets is associated with a number of different problems among female children up to 18 years [1–5]. Bladder problems that manifest during childhood may predict problems such as overactive bladder (OAB), urinary tract infections (UTIs), or incomplete bladder emptying in adulthood [6, 7]. Insufficient privacy, lack of peace and quiet and essential hygiene products, and dirty conditions may result in the desire to void be ignored, thus causing infrequent voiding or low fluid intake [8, 9]. Studies show that urinary incontinence (UI) and other lower urinary tract symptoms (LUTS) are common in women aged 25–90 years and tend to increase with age [10–16]. Wang and Palmer conducted a concept analysis of women’s toileting behavior related to urinary elimination (5915 persons) [17]. Their result showed that urinary elimination can be defined as voluntary actions related to the physiological event of emptying the bladder, which comprises specific attributes, including voiding place, time, position, and style. This behavior also depended on physical and social environments (Fig. 1). From this theory, a questionnaire was developed and validated as the Toileting Behavior scale (TB scale) [18].

Understanding toileting behavior and symptom onset seems to be important for understanding female LUTS [17]. There is, however, a lack of studies on women aged 18–25 years. The question is whether the voiding pattern (place, time, position, and style), established during elementary and secondary school routines, continues among younger women who have finished school. The aim of the study was to investigate toileting behavior and prevalence of LUTS based on the hypothesis that there are correlations between toileting behavior and LUTS among younger women (aged 18–25 years). A further aim was to validate the Swedish version of the TB scale.

## Materials and methods

This was a quantitative descriptive design study using two assessment tools: the International Consultation on Incontinence Questionnaire – Female Lower Urinary Tract Symptoms (ICIQ-FLUTS), and the Toileting Behavior scale (TB scale), together with six background questions about age, height, weight, occupation, parity, and smoking habits. The TB scale consists of 19 questions with five response alternatives each: never (1), rarely (2), sometimes (3), often (4), always (5). It was tested for construct validity and internal consistency. The five subscales were premature voiding (5 items), straining during voiding (4 items), place preference for voiding (4 items), delayed voiding (3 items), and position preference for voiding (3 items) [18]. One question was expressed in a positive direction and was therefore recoded into the same negative direction as the other questions. Therefore, higher scores indicate a more dysfunctional behavior. Permission to use and translate the TB scale into Swedish was given by the constructors. KS, LM and two students at Malmö University did the translation. Experts had the opportunity to comment, and a back-translation was then done with a native English speaker, which resulted in a similar version. The questionnaire was tested in a pilot survey of 100 students at Malmö University and showed a small internal dropout rate, indicating the questions seemed to be easily understood.

ICIQ-FLUTS [19]—tested and translated into many languages including Swedish and with grade A level of validation according to International Continence Society (ICS) recommendations, was used with permission from its developers. It consists of 12 questions with subscales frequency (1–4), voiding (5–7), and urinary incontinence (8–12). Each question had five response alternatives [never (0), occasionally (1), sometimes (2), most of the time (3), all of the time (4)]. The question about day and night frequency and incontinence also had five response alternatives specified in numbers. Higher scores indicate greater impact of individual symptoms. Each question has a bother score from 0 (not at all severe) to 10 (extremely severe). Bother scales are not incorporated in the overall score but indicate the impact of individual symptoms for the patient.

Written information about the purpose of the study and the questionnaires were distributed in November 2014 to 550 women aged 18–25 years randomly selected from the population register in a county in southern Sweden. The information consisted of voluntary participation, the right to withdraw at any time without stating a reason, assurance that all data would be kept confidential, and details of whom to contact in case of questions. A reminder was sent after 3 months. The study was approved by local ethics committee of Lund University with reg. no. 2014/496.

### Statistical analyses

Descriptive statistics were used with mean values and standard deviations (SD). When comparing nonparity with one parity or more, the calculation was performed with Mann–Whitney *U* test, assuming data as being nonnormally distributed. The chi-square test was used when comparing living place between responders and nonresponders. Inspired by van Breda et al. [20], we summarized “sometimes,” “often,” and “always” as “at least sometimes” in the variables in the TB scale. Similarly, we summarised “sometimes,” “most of the time, and “all of the time” as “at least sometimes” in ICIQ-FLUTS variables. These simplifications were also based on the fact that having symptoms sometimes or more often is different from having a symptom rarely or never.

Mean factor scores in the ICIQ-FLUTS and TB scale were estimated by first adding the scores and then dividing them by the number of items in each factor. Question number 18 in the TB scale was transformed to be in the same negative direction as the other items (i.e., higher scores indicating increasing dysfunctional behavior). Correlations between factors of the TB scale and the ICIQ-FLUTS were calculated using Spearman’s rank correlation test and, *p* values < 0.05 were regarded as statistically significant. SPSS version 22.0 was used for all statistical calculations.

The TB scale instrument was tested by a principal component analysis with Varimax rotation, and the factor solution was based on Eigenvalues >1. Factor loadings >0.4 were set as a limit to fit into a factor. Internal consistency was measured with Chronbach’s alpha.

## Results

In total, 550 letters were sent; 26 were returned as address unknown. A total of 173 women responded, which gives a response rate of 33%. Analysis of nonresponders showed that those living in a city had a tendency to a lower response rate than those living outside the cities (*p* = 0.06). The mean age was 21.6 years (range 18–25), and mean body mass index (BMI) 22.9 (range 17–38). In all, 26 were smokers (15.6%). Of the whole sample, 85% (153) were nulliparous and 11.5% were parous (14 were uniparous and six multiparous). In total, 127 women were students in higher education, while 46 were employed. More than 95% completed all questions in the questionnaires.

### TB scale

In all, 87.2% were worried about the cleanliness of public toilets at least sometimes, and 17.4% always avoided using them. To avoid using public toilets, 45.6% always emptied the bladder at home and 11.6% always tried to wait until they came home. In all, 46.5% at least sometimes emptied the bladder without desire at home and just in case. Premature voiding in a public place was reported by 3.6% often or always.

Delayed voiding because of business was reported at least sometimes by 69.9% of the women. The behavior of restraining the desire to void as long as possible occurred at least sometimes in 48.6%, and 46.7% restrained the desire at work or school.

Straining voiding to initiate urinating at least sometimes was reported by 20.3% and during the whole urinating process by 12.7%. To empty the bladder completely, straining while voiding at least sometimes was reported in 38.8% and completely in 42.2%. Most women (97.6%) sat down on the toilet at least sometimes. The behavior of hovering over the toilet occurred at least sometimes in 24.4% of women. Sitting in a squatting position to empty the bladder at least sometimes was reported by 4.2% (Table 1).

**Table 1:** Descriptive data of the Toileting Behavior (TB) scale: results for the five subscales

Questions		Never [*n* (%)]	Rarely [*n* (%)]	Sometimes [*n* (%)]	Often [*n* (%)]	Always [*n* (%)]	At least sometimes [*n* (%)]
Place preference for voiding							
Worry about sanity in public toilets	1	4 (2.3)	19 (11.0)	44 (25.6)	43 (25)	62 (36)	149 (87.2)
Avoid public toilets	2	10 (5.8)	36 (20.9)	51 (29.7)	45 (26.2)	30 (17.4)	126 (72.8)
Empty the bladder at home	3	3 (1.8)	9 (5.3)	2 (14.6)	56 (32.7)	78 (45.6)	136 (92.9)
Try to wait until I come home	4	6 (3.5)	35 (20.3)	70 (40.7)	41 (23.8)	20 (11.6)	131 (76.1)
Premature voiding							
Void without desire:							
At home	5	42 (24.4)	50 (29.1)	48 (27.9)	26 (16.1)	6 (3.5)	80 (46.5)
At work/school	6	78 (45.3)	52 (30.2)	24 (14)	16 (9.3)	2 (1.2)	72 (24.5)
In the home of someone else	7	83 (48.3)	57 (33.1)	20 (11.6)	10 (5.8)	2 (1.2)	32 (18.6)
In a public place	8	121 (70.3)	35 (20.3)	10 (5.8)	4 (2.3)	2 (1.2)	16 (9.3)
“Just in case”, preventive purpose	9	47 (27.3)	45 (26.2)	47 (27.3)	24 (14)	9 (5.2)	80 (46.5)
Delayed voiding							
Try to delay voiding if I’m busy	10	17 (9.9)	34 (19.8)	72 (41.9)	39 (22.7)	10 (5.8)	121 (70.4)
Restrain the desire as long as possible	11	30 (17.4)	59 (34.1)	57 (32.9)	25 (14.5)	2 (1.2)	84 (48.6)
Restrain the desire at work/school	12	34 (19.7)	58 (33.5)	54 (31.2)	19 (11.0)	8 (4.5)	81 (46.7)
Straining voiding							
To initiate the urinating	13	91 (52.6)	47 (27.2)	29 (16.8)	5 (2.9)	1 (0.6)	35 (20.3)
During the whole urinating process	14	111 (64.2)	40 (23.1)	18 (10.4%)	3 (1.7)	1 (0.6)	22 (12.7)
To empty the bladder completely	15	68 (39.3)	38 (22)	42 (23.4)	15 (8.7)	10 (5.8)	67 (38.8)
To empty the bladder faster	16	56 (32.4)	44 (25.4)	56 (32.4)	14 (81)	3 (1.7)	73 (42.2)
Position preference for voiding							
Sit down on the seat	17	1 (0.6)	3 (1.7)	7 (4.)	33 (19.1)	129 (74.6)	169 (97.6)
Hover over the toilet	18	70 (40.7)	60 (34.9)	33 (19.2)	5 (2.9)	4 (2.3)	42 (24.4)
Squat on the toilet	19	153 (89.4)	11 (6.4)	2 (1.2)	3 (1.8)	2 (1.2)	7 (4.2)

Mean dropout for each question was 0.73 (range 0–2)

### ICIQ-FLUTS

Thirty-two participants (19.3%) reported nocturia two or more times. Concerning daytime micturition frequency: 50.9% of women voided more than nine times a day, 2.9% voided one to six times a day, and 46.2% voided seven to eight times a day: 34.2% reported having urgency and 21.1% pain at least sometimes. Hesitancy, straining, and intermittency at least sometimes was reported by 23.6, 22, and 13.9%, respectively. Involuntarily loss of urine was reported by 35.9%: 27.2% once or less per week, 6.4% two or three times per week, and 2.3% once per day. None reported incontinence several times a day. Women who reported having any type of incontinence responded as follows: 32.5% (urge); 47.1% (stress), 23.6% (mixed), and 11.6% (unexplained). One women reported nocturnal enuresis (Table 2).

**Table 2:** Prevalence of lower urinary tract symptoms (LUTS) among younger women

Variable	Bother score	Never n (%)	Rarely n (%)	Sometimes n (%)	Often n (%)	Always n (%)	At least sometimes n (%)
Frequency (4)	1.54						
Nocturia*^,a^	1.08	69 (41.3)	66 (39.5)	23 (13.8)	8 (4.8)	1 (0.6)	32 (19.3)
Urgency*	1.72	46 (27.5)	64 (38.3)	38 (22.8)	16 (9.6)	3 (1.8)	57 (34.2)
Pain*	1.33	100 (60.2)	31 (18.7)	24 (14.5)	7 (4.2)	4 (2.4)	35 (21.1)
Daytime^b^	2.04	5 (2.9)	80 (46.2)	79 (45.7)	8 (4.6)	1 (0.6)	88 (50.9)
Voiding (3)	1.28						
Hesitancy	1.10	60 (34.7)	72 (41.6)	31 (17.9)	7 (4.0)	3 (1.7)	41 (23.6)
Straining	1.09	98 (56.6)	37 (21.4)	32 (18.5)	3 (1.7)	3 (1.8)	38 (22)
Intermittency	0.75	107 (61.8)	42 (24.3)	17 (9.8)	6 (3.5)	1 (0.6)	24 (13.9)
Incontinence (5)	0.59						
Urge	0.98	117 (67.6)	34 (19.7)	20 (11.6)	1 (0.6)	1 (0.6)	22 (12.8)
Frequency, leakage	1.00	111 (64.2)	47 (27.2)	11 (6.4)	4 (2.3)	0	15 (8.7)
Stress	1.12	90 (52.9)	52 (30.6)	24 (14.1)	4 (2.4)	0	28 (16.5)
Unexplained	0.40	152 (88.4)	16 (9.3)	4 (2.3)	0	0	4 (2.3)
Nocturnal enuresis**	0.30	163 (99.4)	1 (0.6)	0	0	0	0 (0)

Mean dropout rate was 2.7 (range 0–9); questions 4–11 had a mean dropout of 0.4 (range 0–2)

*Mean dropout 6.3 (range 6–7)

** Mean dropout 9

^a^Frequency: never (0), rarely (1), sometimes (2), often (3), always (≥4)

^b^Frequency: never (1–6), rarely (7–8), sometimes (9–10), often (11–12), always (≥13)

In relation to maximum scores, the most dysfunctional behaviors were place preference (14.5/20), delayed voiding (7.9/15), and premature voiding (10/25). The parity group of 20 women had significantly higher scores than nulliparous (143) in place preference and delayed voiding. Frequency and voiding had the highest urinary symptoms scores, and significantly higher scores were seen in all dimensions in the parity group (Table 3).

**Table 3:** Toileting Behavior (TB) scale and the International Consultation on Incontinence Questionnaire Female Lower Urinary Tract Symptoms (ICIQ-FLUTS) (mean and in factors)

Toileting behavior	Maximum	Mean (SD)	Mean (SD)	Mean (SD)	*P* value*
		Whole sample (*n* = 173)	Nulliparous (*n* = 146)	Parity (*n* = 20)	
Place preference for voiding	20	14.5 (3.4)	14.1 (3.4)	16.4 (2.3)	0.003
Premature voiding	25	10.0 (4.0)	9.8 (3.9)	11.1 (4.3)	0.180
Delayed voiding	15	7.9 (2.5)	7.8 (2.3)	9.0 (3.2)	0.024
Straining voiding	20	7.6 (3.2)	7.5 (3.1)	8.7 (3.5)	0.153
Position preference for voiding	10	3.3 (1.4)	3.2 (1.8)	3.4 (1.5)	0.751
ICIQ-FLUTS					
Frequency	16	5.3 (2.6)	5.0 (2.6)	6.8 (2.0)	0.000
Voiding	12	2.2 (2.3)	2.0 (2.0)	3.6 (3.1)	0.023
Incontinence	20	1.8 (2.2)	1.5 (2.1)	3.6 (2.5)	0.000

Question about pregnancy, internal dropout = 7

*Difference between nulliparous and parous calculated with Mann–Whitney *U* test

### TB scale correlations to ICIQ-FLUTS

Spearman’s correlation factor was slightly positive for all investigated parameters when the TB scale was compared with the ICIQ-FLUTS (0.060–0.620). The behavior of straining had the strongest correlation to frequency and voiding: (0.326) and (0.620), respectively. The strongest *p* value was found for the correlation between TB subscales straining to void and premature voiding to all ICIQ-FLUTS subscales (*p* < 0.001–0.004). Four TB subscales —place preference for voiding, premature voiding, delayed voiding, and straining voiding—were significantly correlated with the ICIQ-FLUTS subscales frequency and voiding (*p* < 0.0001–0.030). All TB subscales but place preference were significantly related to ICIQ-FLUTS incontinence subscale (*p* < 0.001–0.043) (Table 4).

**Table 4:** Correlation between Toileting Behavior (TB) scale and International Consultation on Incontinence Questionnaire short form (ICIQ-SF)

ICIQ-FLUTS	Toileting behavior				
	Place preference for voiding	Premature voiding	Delayed voiding	Strain for voiding	Position for voiding
Frequency					
Spearman’s rank correlation	0.224	0.226	0.223	0.310	0.134
* P* value	0.004	0.003	0.004	<0.001	ns*
Voiding					
Spearman’s rank correlation	0.169	0.250	0.315	0.646	0.071
*P* value	0.028	0.001	<0.001	<0.001	ns*
Incontinence					
Spearman’s rank correlation	0.031	0.154	0.179	0.212	0.072
*P* value	ns*	0.050	0.023	0.007	ns*

NS nonsignificant (i.e., *p* value >0.05

Principal component analysis with Varimax rotation revealed five underlying factors that explained 66% of the variance. This factor solution was in agreement with the developers of the questionnaire. Cronbach’s alpha values within each factor were satisfactory (0.71–0.84) and similar to the original, except for position preference, which had a Cronbach’s alpha of 0.54 compared with 0.73 in the original [18] However, the Swedish version was supplemented with one question in this category (Table 5).

**Table 5:** Principal component analysis of Toileting Behavior (TB) scale to test construct validity and results of reliability test (Cronbach’s alpha values)

Questions		Premature voiding	Place preference	Straining voiding	Delayed voiding	Position preference
Worry about sanity in public toilets	1		0.763*			
Avoid public toilets	2		0.881*			
Empty the bladder at home	3		0.755*			
Try to wait until I come home	4		0.706*			
Void without desire:						
At home	5	0.629*				
At work/school	6	0.860*				
Home of someone else	7	0.831*				
A public place	8	0.827*				
Just in case (preventive)	9	0.758*				
Try to delay voiding if I’m busy	10				0.857*	
Restrain the desire as long as possible	11				0.819*	
Restrain the desire at work/school	12				0.545*	
Strain to initiate urinating	13			0.823*		
Strain during the whole urinating process	14			0.889*		
Strain to empty the bladder completely	15			0.836*		
Strain to empty the bladder faster	16			0.645*		
Sit down on the seat	17					0.643*
Hover over the toilet	18					0.715*
Squat on the toilet	19					0.751*
Eigen value		3.868	3.048	2.442	1.691	1.483
Explained variance (%)		20.36	16.04	12.86	8.90	7.81
Total 66.0%						
Cronbach’s alpha value		0.841	0.809	0.822	0.714	0.542

*Factor loadings: values >0.5 are considered practically significant [21]

## Discussion

The hypothesis in our study was corroborated, as there were significant relations between toileting behavior and LUTS among younger women. This is an interesting finding and could have important clinical implications. For example, Moore et al. found that residual urine increased by 149% in those who hovered over the toilet instead of sitting in a relaxed position [22]. However, the population in that study were older (25–81 years). In our study, most women usually sat on the toilet. The most frequent behaviors were within the factors place preferences (i.e., avoiding toilets) and different strategies to delay voiding. Most study participants were students or worked and thus could avoid public toilets, even though a majority were worried about the cleanliness of public toilets in, e.g., shopping centers, petrol stations, restaurants, or pubs.

There seems to be a lack of knowledge among young and middle-aged women about a normal way of emptying the bladder; for instance, avoiding straining when voiding [23]. It is not clear what impact straining has on voiding dysfunction [23–25]. However, symptoms connected to the emptying phase often coexist with symptoms correlated to the storage phase. Behavior when emptying the bladder could be important for understanding symptom origin and choosing the appropriate intervention for the LUTS patient. An easy intervention is to teach the patient a relaxed, optimal way of emptying the bladder. Symptoms due to a dysfunctional emptying pattern may be reduced with correction of the pattern. Therefore, this study may indicate the need to investigate a patient’s voiding pattern to obtain an accurate picture and manage symptoms that may be due to dysfunctional toileting behavior.

Surprisingly, many young women reported urinary symptoms when using the definition of having symptoms at least sometimes or more often. However, there is a lack of definition for response alternatives, which can be confusing both for the responder and interpreter. On the other hand, they also reported having symptoms more frequently than occasionally. Our interpretation, therefore, is that they actually have symptoms, which is why data are summarized in this way. According to the definition of the ICS, for instance, urinary incontinence is “any urinary leakage,” with no definition of how frequent it is [26]. Urinating nine times or more during the day, urgency, hesitancy, and straining were the most common symptoms. Van Breda et al. performed a study using ICIQ-FLUTS assessing 159 presumably healthy medical students aged 18–30 years [20]. They also summarized the alternatives in that way. Comparing our results showed that our group had more symptoms in all the investigated parameters. For example, urgency at least sometimes was reported by 34.2% of our group compared with 14.5% in their group. Pauwels et al. also found that middle-aged women had large bladder capacity and high bladder compliance, indicating that women tended to postpone toileting as long as possible [27]. Bladder pain was also quite common (21%), but it is not clear what sort of pain. One explanation could be that cystitis problems in this age group are common, or irritative symptoms are from genitalia after sexual activity. Generally, our results showed higher prevalence rates compared with other studies, indicating that responders were women with more problems.

Higher scores in ICIQ-FLUTS subscales suggest a greater impact of separate symptoms for the patient, but there is no further information about how to interpret the summarized scores, as in the International Prostate Symptom Score (IPSS), in which symptom scores are classified as mild (0–7), moderate (8–19), and severe (20–35) for comparing symptom progression and severity and determining treatment [28].

The significant difference in all dimensions of urinary symptoms, not only incontinence, in the parity group with higher scores is interesting and to our knowledge not reported previously. However, the parity group was small, and this result must be viewed with caution. Parous women with LUTS often have increased laxity in passive-support structures, a deficit in the onset of the pelvic floor and strength to support the bladder neck when intra-abdominal pressure increases. Women with LUTS are also more likely to use straining voiding and for a longer time than healthy women with no symptoms [17]. The way straining is performed and whether LUTS is or is not present seem to be important. Structural changes due to childbirth might alter lower-urinary-tract function and behavior—or the impact of a behavior. More studies are needed in this regard.

Construct validity and reliability of the translated Swedish version of the TB scale showed similar results to the original. However, no other aspects of validity were tested, e.g., comparing results objective measures, which is difficult in a postal questionnaire survey. The TB scale was easy to use and questions well understood, as the internal dropout rate was low; > 95% answered all questions. Factors in the questionnaire’s subscales assessing toileting behavior made it possible to use to analyze relationships, in this case with ICIQ-FLUTS, even though this was not in the aim of instrument validation. Furthermore, it is feasible to assume that the translated version of the TB scale would be an aid in clinical practice to evaluate and modify toileting behavior, thus promoting good bladder health. To strengthen its generalizability, further testing is needed in community-dwelling and hospitalized populations.

Attaining the sample of young women seemed to be difficult, as the response rate was low. This must be taken into consideration when interpreting the results. Also, many questionnaires were returned as address unknown. One can assume that those who answered may have more problems than those who did not. However, that issue remains unknown. Dropout analysis showed a tendency of lower response rate among those living in the cities compared with those living in the country, but it is unclear what consequences that issue may have on results. Using e-mail instead of the postal system may have reached more women, but such data is not available in the population register in Sweden. Another option was to use Facebook or other social media, but a representative sample would not have been reached in that case either. Even though the intention was to have a representative sample from the population in this age group, we obtained a rather large sample and can therefore make some conclusions about young women and their urinary symptoms relative to toileting behavior. Studies among women in this age group are few, usually among selected groups only, but are not representative of the general population. However, the strength in this study was the finding of a relation between urinary symptoms and toileting behavior among younger women. To our knowledge, this has not yet been described in the literature.

## Conclusion

Lower urinary tract symptoms were quite common in this group of young women. Toileting behaviors were also significantly related to urinary tract symptoms. Thus, the Toileting Behavior scale (TB scale) was useful for assessing this population, and the translated Swedish version showed good validity and reliability. Further research about the impact of toileting behavior is needed to understand the origin and development of LUTS and its treatment.
